# MKL-1 is a coactivator for STAT5b, the regulator of Treg cell development and function

**DOI:** 10.1186/s12964-020-00574-1

**Published:** 2020-07-09

**Authors:** Yuan Xiang, Jun Wang, Jia Peng Li, Wei Guo, Feng Huang, Hui Min Zhang, Han Han Li, Zhou Tong Dai, Zi Jian Zhang, Hui Li, Le Yuan Bao, Chao Jiang Gu, Kun Chen, Tong Cun Zhang, Xing Hua Liao

**Affiliations:** 1 Institute of Biology and Medicine, College of Life and Health Sciences, Wuhan University of Science and Technology, Wuhan, Hubei 430081 PR China; 2 Shenzhen Ritzcon Biological Technology Co., LTD, Shenzhen, Guangdong 518000 PR China; 3 Medical School, Liaocheng University, No.1 Hunan Road, Liaocheng, 252000 China; 4 Key Laboratory of Industrial Fermentation Microbiology, Ministry of Education and Tianjin, College of Biotechnology, Tianjin University of Science and Technology, Tianjin, PR China 300457

**Keywords:** MKL-1, STAT5b, Treg, ITP

## Abstract

**Background:**

Foxp3^+^CD4^+^ regulatory T cells (Treg) constitutes a key event in autoimmune diseases. STAT5b is the critical link between the IL-2/15 and FOXP3, the master regulator of Treg cells.

**Methods:**

The CD3^+^T cell and Foxp3^+^CD4^+^ regulatory T cells were overexpressioned or knockdown MKL-1 and STAT5a and tested for Treg cell development and function. Direct interaction of MKL-1 and STAT5a were analyzed by coimmunoprecipitation assays, Luciferase assay, Immunofluoresence Staining and Yeast two-hybrid screening. The effect of MKL-1 and STAT5a on the Treg genes expression was analyzed by qPCR and western blotting and Flow cytometry.

**Results:**

However, the molecular mechanisms mediating STAT5b-dependent Treg genes expression and Treg cell phenotype and function in autoimmune diseases are not well defined. Here, we report that the MKL-1 is a coactivator for the major Treg genes transcription factor STAT5b, which is required for human Treg cell phenotype and function. The N terminus of STAT5b, which contains a basic coiled-coil protein–protein interaction domain, binds the C-terminal activation domain of MKL-1 and enhances MKL-1 mediated transcriptional activation of Treg-specific, CArG containing promoters, including the Treg-specific genes Foxp3. Suppression of endogenous STAT5b expression by specific small interfering RNA attenuates MKL-1 transcriptional activation in cultured human cells. The STAT5b–MKL-1 interaction identifies a role of Treg-specific gene regulation and regulated mouse Treg cell development and function and suggests a possible mechanism for the protective effects of autoimmune disease Idiopathic Thrombocytopenic Purpura (ITP).

**Conclusions:**

Our studies demonstrate for the first time that MKL-1 is a coactivator for STAT5b, the regulator of Treg cell development and function.

Video abstract

## Background

Foxp3^+^CD4^+^ regulatory T cells (Treg) have a crucial role in controlling CD4^+^ T-cell activation, proliferation, and effector function [1]. Treg constitutes a key event in the development of autoimmune diseases such as Idiopathic Thrombocytopenic Purpura (ITP) [2]. However, the molecular mechanisms underlying the function and stability of the Treg compartment have not been fully elucidated.

Megakaryoblastic leukemia 1 (MKL-1), was initially identified acute megakaryoblastic leukemias in infants and young children [3–5]. MKL-1 is a myocardin-related coactivator of the serum response factor (SRF) transcription factor, which has an integral role in differentiation, migration, and proliferation in different types of tissues or cells [6]. Functions for MKL-1 have been characterized in embryonic stem cells, fibroblasts, smooth muscle cells, and neurons [7–9]. Recent studies have shown that MKL-1 in megakaryocyte differentiation is mediated by association with SRF [10]. Based on these findings, we have examined the effects of the MKL-1 in Treg cell phenotype and function.

The signal transducer and activator of transcription (STAT) 5b plays indispensable roles in immunodeficiencies, autoimmunities, cancers [11, 12]. The importance of STAT5b for the in vivo accumulation of Treg with immunoregulatory function has been demonstrated [12, 13]. Recent studies demonstrated that STAT5 was the critical link between the interleukin-2 (IL-2) /15 and FOXP3 [14, 15], the core gene of Treg cells. Low-dose IL-2 promoted phosphorylation of STAT5, Treg survival and CTLA-4-dependent function in autoimmune [16]. Binding of IL-2 to IL-2RA (CD25) on Treg led to phosphorylation of STAT5, resulting in up-regulation of Foxp3 [17, 18] and functional surface markers. The specific role that STAT5b plays in the pathogenesis of the autoimmuned diseases suggested that the transcription factor might have potential as a novel diagnostic and/or therapeutic target in some disease settings.

The current study uncovers the mechanistic basis by which MKL-1-STAT5b axis potentiates Treg cells, its relevance to human biology, and explores the clinical implications of these findings using an experimental autoimmune disease of ITP.

## Materials and methods

### Reagents

Recombinant human IL-2 protein was obtained from R&D Systems. (Emeryville, CA, USA) Antibodies to Foxp3, CD25, MKL-1, pSTAT5 and STAT5 were purchased from Cell Signaling Technology (CST, Boston, Massachusetts, USA) and BD (New York, New Jersey, USA); Myc, Flag and HA antibody was purchased from proteintech (Wuhan, China).

### Cell isolation

For Peripheral blood mononuclear cells (PBMC), we used MACSprep™ PBMC Isolation Kit (Miltenyi Biotec, Germany) to perform the separation under the manufacturer’s instructions. For CD3^+^ T cells, we used CD3 MicroBeads, human (130–050-101, Miltenyi Biotec, Germany) to isolate according to the manufacturer’s instructions. For Treg, we used MACSxpress Whole Blood Treg Isolation Kit, human (130–109-557, Miltenyi Biotec, Germany) for isolation.

### Cell culture and transient transfection

CD3^+^ T cells cover in Serum -free Medium (T cells) (Gibco, Grand Island, New York, USA) then treated IL-2 for 48 h at 37 °C in a 5% CO_2_ incubator. For transient expression experiments with CD3^+^ T cells were transfected for 3 h with 2 g of plasmids by using 8uL of Lipofectamine 2000 and 8uL of Plus reagent (Invitrogen, Carlsbad, California, USA). Treg cells were expanded using the Treg Expansion Kit, human (Miltenyi Biotec, Germany) according to the previously reported method [19, 20]. In briefly, the isolated cells were cultured in TexMACS™ GMP Medium added with MACSiBead™ Particles pre-loaded with CD3 and CD28 antibodies at a certain ratio, and stimulated with the appropriate concentration of IL-2 under the manufacturer’s instructions. The CD4^+^ CD8^−^Foxp3^+^CD44^lo^CD62L^hi^CD25^hi^ CTLA4^lo^ population in Treg is defined as cTreg, The CD4^+^ CD8^−^Foxp3^+^CD44^hi^CD62L^lo^CD25^lo^ CTLA4^hi^ population in Treg is defined as eTreg according to previous reports [21, 22].

### T cell suppression assay

For Treg suppression assay, autologous CD4^+^CD25^−^ responder T cells (Tresp cells) were used as responder cells. Tresp cells were stained with eFluor670 dye and mixed with expanded Tregs at the ratio of 1:1, 2:1 and 4:1 in culture media, and stimulated with anti-CD3 mAb (HIT3a, BD Biosciences) and IL-2 (50 U mL^− 1^). After 4 days, cells were collected and analyzed by flow cytometry. Suppression index was calculated by the percentage of CD8 cells in responder cells that underwent division. Suppression index was calculated using following equation: (Tresp proliferation without Treg−Tresp proliferation with Treg)/Tresp proliferation without Treg according to previous reports [21].

### Immunofluoresence staining

Cells after treatment were fixed in 4% paraformaldehyde for 20 min, and then blocked with normal goat serum for 20 min at room temperature. Then, rabbit MKL-1 (Abcam, Cambridge, UK, ab49311) and mouse STAT5b (Abcam, Cambridge, UK, ab178941) antibodies were added and incubated in a humid chamber overnight. After washing with PBS twice, cells were incubated with appropriate secondary antibodies (fluorescein isothiocyanate (FITC)-goat anti-rabbit (Servicebio, China, GB22303), TRFITC-goat anti-mouse) for 30 min at 37 °C. After washing with PBS, the samples were observed by laser scanning confocal microscopy (Olympus, Japan). 4′,6-diamidino-2-phenylindole (DAPI) stain (blue) highlights the total nuclei.

### Luciferase reporter assays

Luciferase assays were performed as described previously [23]. After transfection for 24 h, luciferase activity was measured by a Synergy 4 (Promega, Madison, MA, USA). Transfection affeciencies were normalized to total protein concentration of each luciferase assay preparation. All experiments were performed at least three times with different preparations of plasmids and primary cells, producing qualitatively similar results. Data in each experiment are presented as the mean ± standard deviation of triplicates from a representative experiment.

### Quantitative real-time PCR (qRT-PCR)

We extracted total RNA using the trizol reagent (Invitrogen, Carlsbad, CA, USA) according to the manufacturer’s instructions. The RNA content was measured with an spectrophotometer (Implen, Munich, Germany). For qRT-PCR, we used sybr premix (Takara, Dalian, China) in a real-time PCR cfx96 system (Bio-Rad, USA). The primer sequences used for qRT-PCR were as follows: MKL1 forward: 5′-CAAACGGAAGATTCGTTCCCG-3′, reverse: 5′-TTGAGGTCATCGGCTAGTCTG-3′; STAT5 forward: 5′-CAGAACACGTATGACCGCTG-3′, reverse: 5′-CTGGAGAGCTACCATTGTTGG-3′; FOXP3 forward: 5′-GTGGCCCGGATGTGAGAAG-3′, reverse: 5′-GGAGCCCTTGTCGGATGATG-3′; CD25 forward: 5′- GTGGGGACTGCTCACGTTC-3′, reverse: 5′-CCCGCTTTTTATTCTGCGGAA-3′; GAPDH forward: 5′-GGAGCGAGATCCCTCCAAAAT-3′, reverse: 5′- GGCTGTTGTCATACTTCTCATGG -3′. GAPDH was used for loading control. The primer sequences used to amplify the recovered product of protein co-immunoprecipitation are as follows: CArG forward: 5′-CTGTTAAACACCTGCTGTTCAC-3′, reverse: 5′- GCCTGATGGCCTGGCTTTG-3′; CAS forward: 5′-TCAGAGGCCCCTGTCTCTG-3′, reverse: 5′-CCAGAGATATAGGAGGCAAAC-3′.

### siRNA design and transient transfection

SiRNAs for STAT5 were designed with BLOCK-iT RNAi designer (www.invitrogen.com). The siRNAs and a scramble negative control (NC) were chemically synthesized (Ribobio, Guangzhou, China). The transfection of siRNA into CD3^+^ T cells was performed with Lipofectamine 3000 (Invitrogen, Carlsbad, California, USA) following the manufacturer’s protocol for CD3^+^ T cells.

### Immunoprecipitation and Western blotting analysis

Cells were harvested in lysis buffer (Beyotime, Shanghai, China) and equal amounts of proteins were incubated with a specific antibody overnight at 4 °C with gentle rotation. Protein A/G Plus-agarose beads (Santa Cruz Biotechnology, Dallas, Texas, USA) were used to pull down the antibody complexes. Immune complexes were then separated by SDS/PAGE and analyzed by Western blotting. The primary antibody used was as follow: MKL-1 (Abcam, Cambridge, UK, ab49311), STAT5b (Abcam, Cambridge, UK, ab178941), p-STAT5b (Abcam, Cambridge, UK, ab52211), FOXP3 (Abcam, Cambridge, UK, ab22510), MYC-tag (Proteintech, China, 66,004–1-Ig), Flag-tag (Proteintech, China, 20,543–1-AP).

### Yeast two-hybrid screening

The protein interactions were screened by the yeast transcription activator protein upstream activation sequence (GAL4-UAS) system (Clontech, Japan). Fused the truncated mutant human MKL-1 (MKL1-M) to the Gal4 DNA-binding domain (Gal4 BD), fused the Jurkat cells cDNA library (HKL160269_LJP) to the Gal4 activation domain (Gal4 AD) (pGADT7-library). After confirming that there was no self-activation of MKL1-M bait clone, co-transformated bait plasmid and library prey plasmids to AH109 yeast strain and coated onto SD-TL-deficient (SD-TL:-trp, −leu) plates. Subsequently, the blue clones were patched out onto higher stringency SD-TLHA (SD-TLHA:-trp, −leu, −his, −ade) plates. Those colonies that were still blue indicating that they were likely to be positive hits, which were amplified using *E. coli* transformation, PCR or both and sequenced.

### IL-2 binding assay

IL-2 was biotinylated using EZ-Link Sulfo-NHS-Biotin (Thermo Scientific) and incubated with Treg treated with AG490 orY27632 or medium. After 30 min of stimulation, Treg were incubated with biotinylated IL-2 followed by staining with streptavidin.

### Statistical analysis

Data were shown as mean ± SD for 3 or 6 separate experiments. Differences were analyzed by Student’s t test. Values of *p*<0.05 were considered statistically significant.

## Results

### MKL-1 and STAT5b are high expressed in Treg cells

**Fig. 1:**
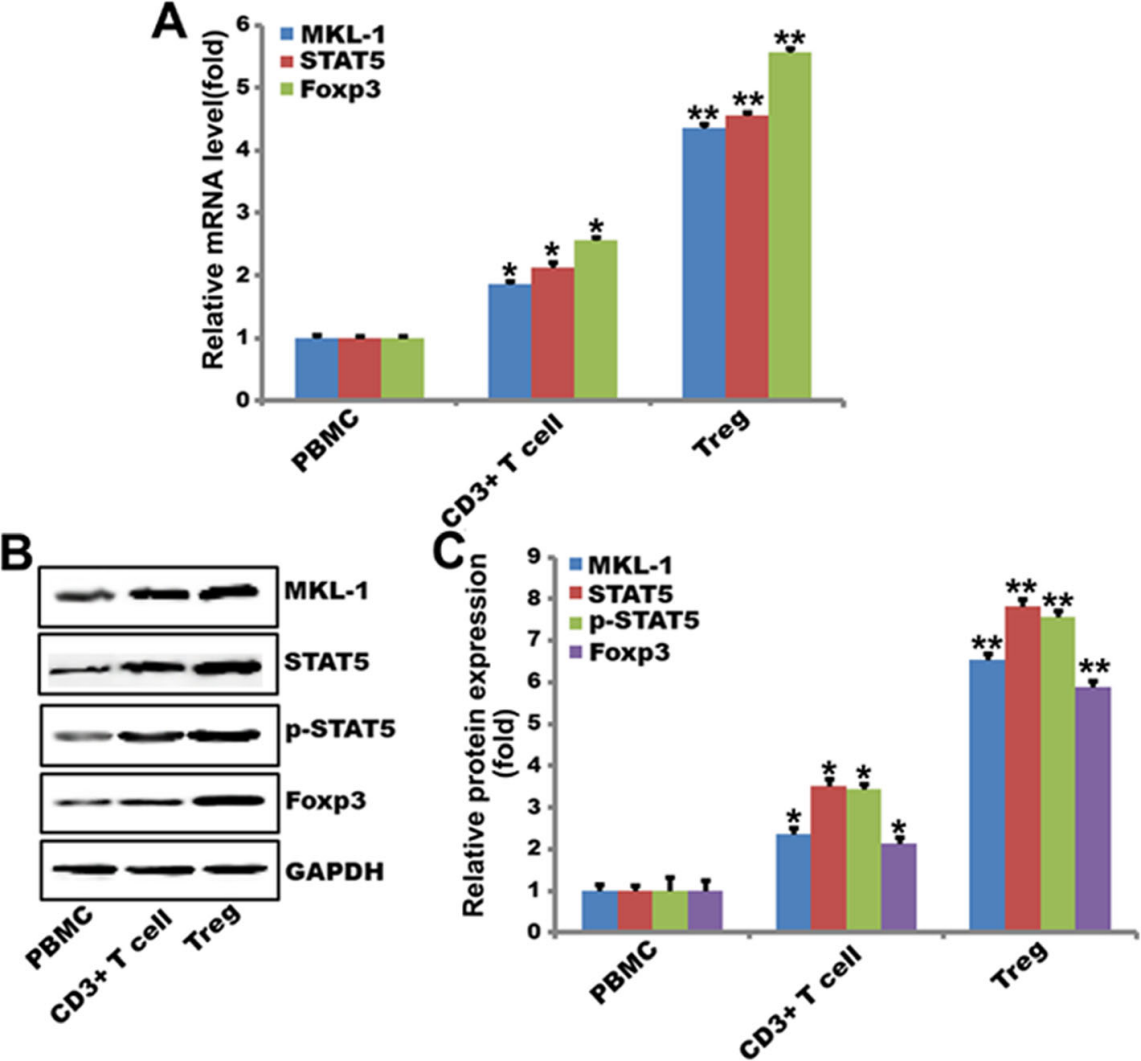
MKL-1 and STAT5b are high expressed in Treg cells. **a** QPCR analysis of MKL-1 and STAT5b mRNA level in PBMC, CD3^+^T cells and Treg cells. GAPDH is the loading control. **p < 0.01, *p < 0.05. *n* = 3; **b** Western blot analysis of MKL-1 and STAT5b expression in PBMC, CD3^+^T cells and Treg cells. Data were quantified using Quantity One software. GAPDH is the loading control. **p < 0.01, *p < 0.05. *n* = 3

The expression levels of MKL-1, STAT5b and Foxp3 transcripts in human PBMC, CD3^+^ T and Treg cells were detected by qPCR (Fig. 1a), with the highest expression levels human Treg cells. Expression of MKL-1, STAT5b, p-STAT5b and Foxp3 proteins was also detected in human PBMC, CD3^+^ T and Treg cells (Fig. 1b). Similarly, MKL-1, STAT5b and Foxp3 had the highest levels in human Treg cells (Fig. 1c).

### Over-expression MKL-1 and STAT5b increase the number of Treg in CD3^+^ T cells and enhance the Treg markers expression

Transfected MKL-1 or STAT5b (causing over-expression MKL-1 and STAT5b; Supplemental Fig. 1A) alone resulted in increased the number of Treg cells in CD3^+^ T cells. Cotransfection of plasmids encoding full-length STAT5b with MKL-1 synergetic increases the number of Treg cells (Supplemental Fig. 1B). MKL-1 increased the number of Treg cells by 2.2-fold, and STAT5b alone increased the number of Treg cells by 3.2-fold. Cotransfected MKL-1 and STAT5b increased the number of Treg cells by 7.3-fold (Supplemental Fig. 1B). Subsequently, Transfected MKL-1 or STAT5b alone enhanced the expression of Foxp3 and CD25. Cotransfection STAT5b with MKL-1 synergetic induced the mRNA and protein level of Foxp3 and CD25 (Supplemental Fig. 1C-E). Together, these data support that over-expression MKL-1 and STAT5b increase the number of Treg in CD3^+^ T cells and enhance the Treg markers expression.

### Inhibited or knock-down MKL-1 and STAT5b weaken the Treg markers expression

Foxp3 and CD25 are known to be critical for Treg function. To investigate whether an inhibition RhoA-MKL-1 and JAK-STAT5 by Y27632 and AG490 a reduction in MKL-1 and STAT5b indeed translates into suppressed Treg markers mRNA and protein levels, we compared the expression of Foxp3 and CD25 in the presence of control (treated with DMSO) or treated with Y27632 or AG490 in CD3^+^ T cells (Supplemental Fig. 2A, C and E). In treated with AG490 group, reduced of Foxp3 and CD25 mRNA and protein level occurred (Supplemental Fig. 2A, C and D). Similar to that of treated with AG490, treated with Y27632 resulted in reducing Foxp3 and CD25 mRNA and protein level (Supplemental Fig. 2A, C and D). Importantly, cotreated Y27632 and AG490 reduced Foxp3 and CD25 and protein level (Supplemental Fig. 2A, C and D). To test whether the expression of Foxp3 and CD25 in STAT5b-depleted cells still remained dependent on MKL-1, cells were cotransfected with MKL-1 and STAT5b siRNAs. The inhibition of MKL-1 or STAT5b expression resulted in reducing Foxp3 and CD25 mRNA and protein level, respectively. Cotransfected with MKL-1 and STAT5b siRNAs resulted in reducing Foxp3 and CD25 mRNA and protein level (Supplemental Fig. 2B, D and F). Together, these results indicate that inhibited or knock-down MKL-1 and STAT5b weaken the Treg markers expression.

### IL-2 affect the effect MKL-1 and STAT5b on the Treg marker expression

Our data thus far suggested that Treg respond to RhoA-MKL-1 and JAK-STAT5 signaling by maintaining their phenotype and the expression of surface markers. It is well known that IL-2 is required to prevent the development of systemic autoimmune disease [24]. As shown in Supplemental Fig. 3A and B, IL-2 enhances the mRNA and protein level of STAT5b with MKL-1 mediated the induction of Foxp3, respectively. The Foxp3 reporter results show that IL-2 enhances the transcriptional activity of STAT5b with MKL-1 mediated the induction of Foxp3, respectively (Supplemental Fig. 3C). Thus, IL-2 enhanced MKL-1 and STAT5b mediated the induction of Foxp3.

### RhoA-MKL-1 and JAK3-STAT5b signaling stabilizes the activated Treg development and function

**Fig. 2:**
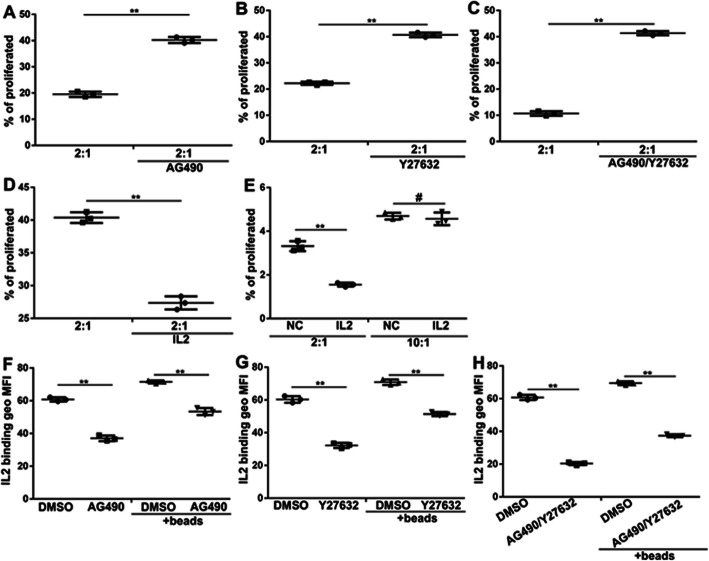
RhoA-MKL-1 and JAK3-STAT5b signaling stabilizes the activated Treg development and function. **a** Percentage of proliferated cells in 2:1 Treg: CD4^+^T cells and treated AG490 for 48 h (*n* = 6, SEM, t test). Data are representative of three experiments; **b** Percentage of proliferated cells in 2:1 Treg: CD4^+^T cells and treated Y27632 for 48 h (*n* = 6, SEM, t test). Data are representative of three experiments; **c** Percentage of proliferated cells in 2:1 Treg: CD4^+^T cells and treated AG490 or Y27632 for 48 h (*n* = 6, SEM, t test). Data are representative of three experiments; **d** and **e** Proliferation stimulated with anti-CD3/CD28 beads for 48 h determined by eFluor670 proliferation dye staining in a 2:1 or 10:1 (Foxp3+ Treg: CD4^+^T cells) ratio with or without 50 ng/mL IL-2 for 48 h (*n* = 6, SEM, t test); **f**, **g** and **h** IL-2 binding by medium and AG490 or Y27632–stimulated Foxp3^+^ Treg in the absence orpresence of CD3/CD28 bead stimulation. (*n* = 6, SEM, t test)

Treg treated with AG490 or Y27632 were able to suppress proliferation of CD4^+^ T cells more efficiently than nopretreated Treg (Fig. 2a and b). Moreover, cotreated AG490 and Y27632 remarkably suppress proliferation of CD4^+^ T cells (Fig. 2c). The CD4^+^ T cells proliferation was induced by IL-2 (2:1 group), whereas the addition of 10 times (10:1 group) more Treg and exogenous IL-2 further increased Treg-mediated suppression of CD4^+^ T cells proliferation in the 2:1 group, but not in the presence of a high number of Treg in the 10:1 group (Fig. 2d and e). Interestingly, AG490–pretreated Treg had no pSTAT5 (Supplemental Fig. 5) and had decreased binding of IL-2 (Fig. 2f). Similar to AG490, treated with Y27632 Treg had decreased binding of IL-2 (Fig. 2g). Moreover, cotreated AG490 and Y27632 remarkably suppress the ability binding of IL-2 (Fig. 2h). These data strongly suggest that Treg exposed AG490 or Y27632 suppress their capacity to binding IL-2 and inhibit CD4^+^ T cell proliferation.

### Ag490 and Y27632 affect the phosphorylation of Foxp3 and nuclear accumulation of Foxp3

Having seen that inhibited or knock-down MKL-1 and STAT5b weaken the Treg markers expression, we wished to determine how these inhibitions of MKL-1 or STAT5b impact the nucleocytoplasmic transport of Foxp3. To address this, we used Western blotting of nuclear extracts and membrane extracts (Supplemental Fig. 4C and D). Importantly, AG490 or Y27632 provoked strong inhibiting nuclear translocation and phosphorylation of Foxp3 (Supplemental Fig. 4 A and B). Furthermore, when AG490 was added together with Y27632, both the phosphorylation of Foxp3 and the overall nuclear MRTF content was lower than treated alone AG490 or Y27632. It is noteworthy that when combined with AG490 or Y27632 markedly inhibited nuclear Foxp3 accumulation. These findings show that Ag490 and Y27632 affect the phosphorylation of Foxp3 and nuclear accumulation of Foxp3 to regulate Treg cell Treg phenotype and function.

### MKL-1 and STAT5b interacted in vivo and vitro

**Fig. 3:**
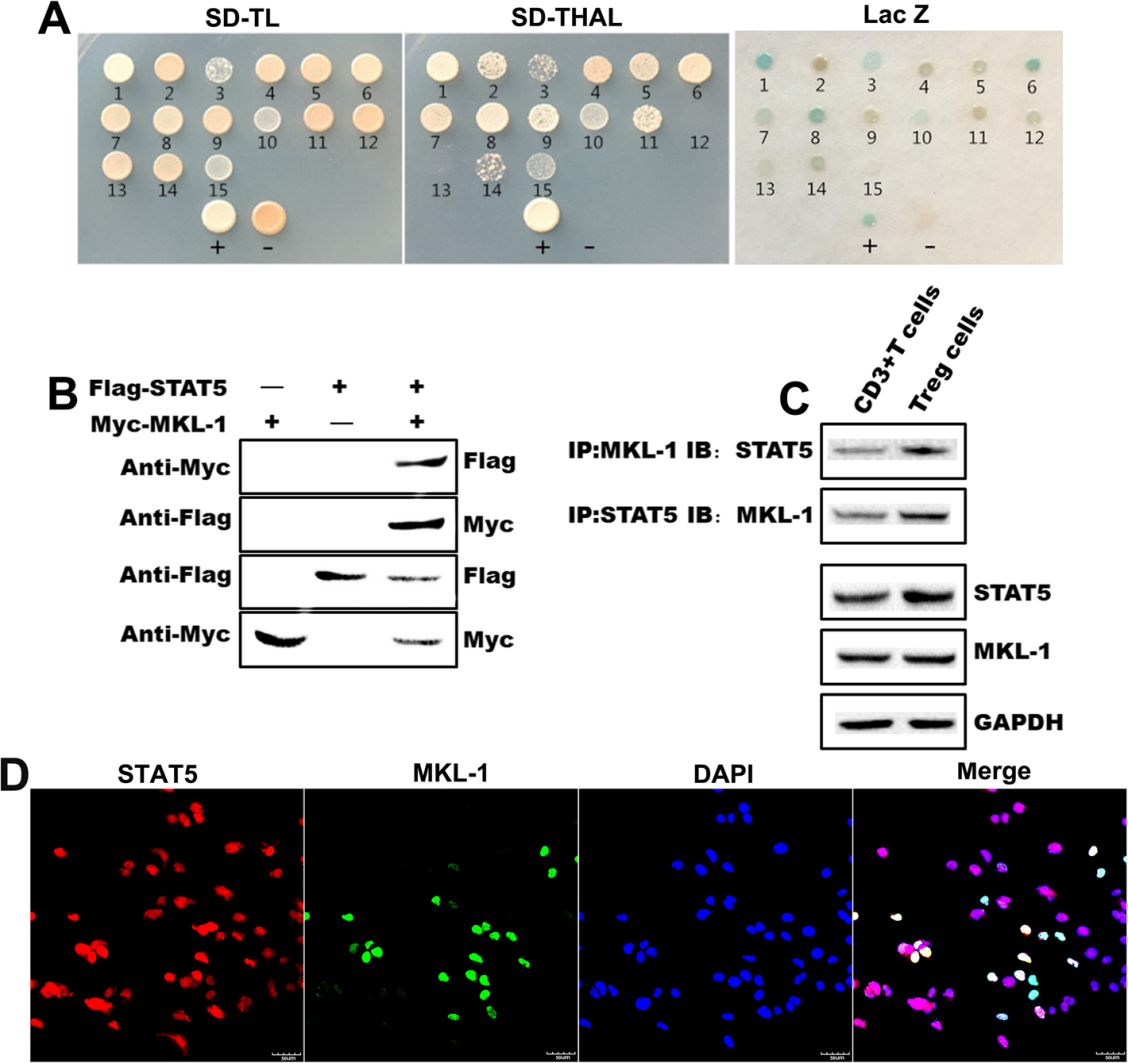
MKL-1 and STAT5b interact in vivo and vitro. **a** Y2H assay to detect the MKL1-STAT5 interaction; **b** and **c** Coimmunoprecipitiation of MKL1 with STAT5 in CD3^+^ T cells and Treg cells; **d** Immunofluorescence assay to detect the MKL1-STAT5 colocalization in Treg cells

Y2H assay results showed that all of the 12 positive clones except the 14th could activate the HIS3 and ADE2 reporter genes, of which 4, 10, and 15 were weakly activated. Only 1, 3, 6, 8, and 10 of the 12 positive clones can activate the LacZ reporter gene (Fig. 3a). These results show that Yeast two-hybrid screen (Y2H) interactions were confirmed that MKL-1 interacted with STAT5b in CD3^+^ T cells.

We hypothesized that MKL-1 and STAT5b influence Treg cell phenotype and function might interact between STAT5b and MKL-1 in Treg cells. So Coimmunoprecipitation assays demonstrated a direct interaction between the STAT5b and MKL-1 in CD3^+^ T cells (Fig. 3b) and Treg cells (Fig. 3c). These data have show that MKL-1 and STAT5b have an interaction in CD3^+^ T cells (Fig. 3b) and Treg cells (Fig. 3c). Immunofluorescence of endogenous MKL-1 and STAT5b from Treg cells cells to colocalization, which express high levels of MKL-1 and STAT5b (Fig. 1a), was also observed (Fig. 3d). The data show that these co-located cells are MKL-1^+^ STAT5^+^ positive Tregs (Fig. 3d).

### MKL-1TAD domain interacts with STAT5b coiled-coil domain

**Fig. 4:**
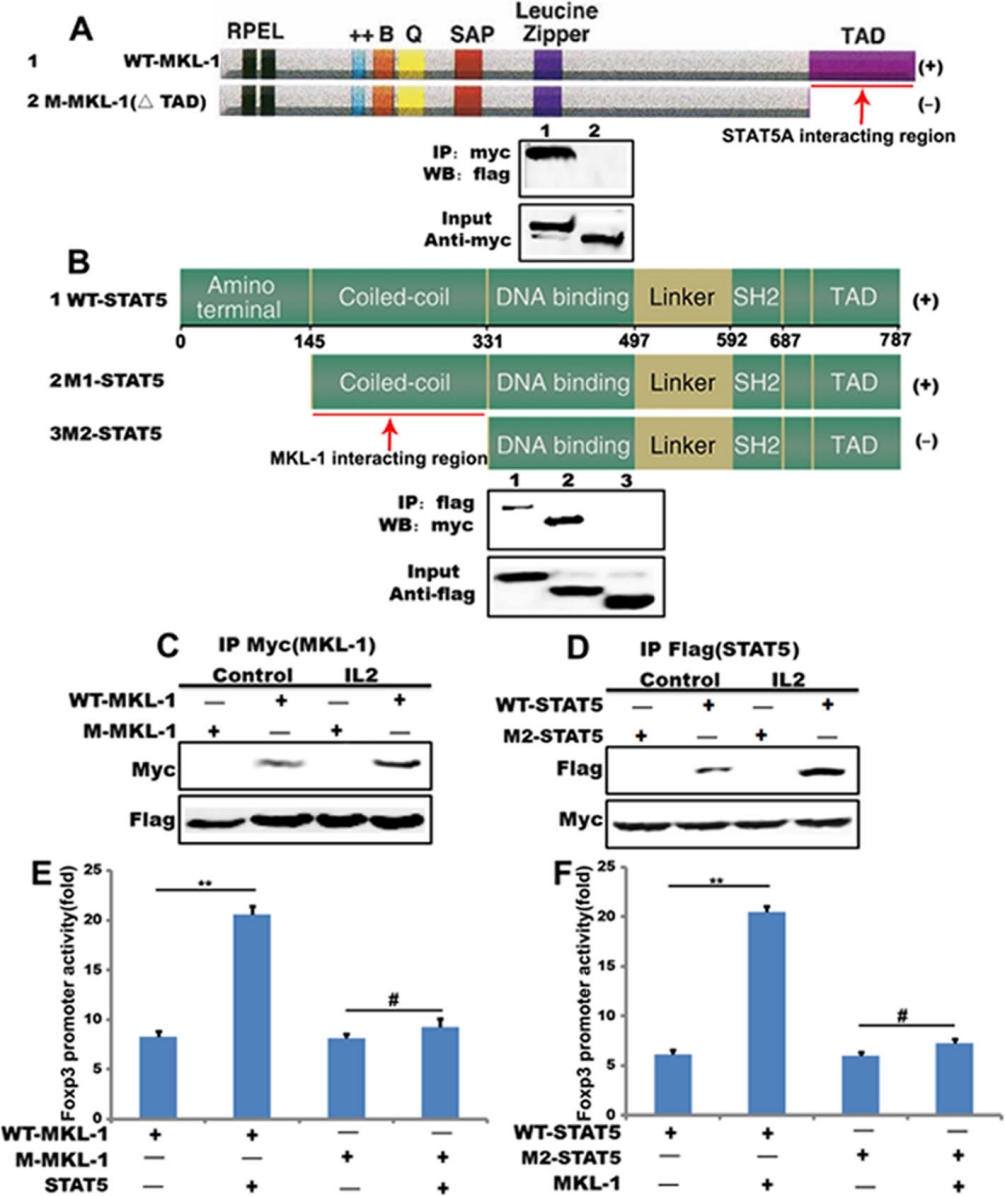
MKL-1TAD domain interacts with STAT5b coiled-coil domain. **a** and **b** Domain structure of MKL-1 and STAT5b. STAT5b interacts with MKL-1from CD3^+^T cells transfected with mammalian Flag-tagged STAT5b and Myc-tagged MKL-1 (1–705) (MKL-1△TAD). MKL-1 interacts with STAT5b from CD3^+^T cells transfected with mammalian Myc-tagged MKL-1 and Flag-tagged STAT5b (145–787) (M1-STAT5b) or Flag-tagged STAT5b (331–787) (M2-STAT5b); **c** The predicted STAT5b-binding site (MKL-1 TAD) is shown in A. This latter region was deleted to generate MKL-1 TAD domain mutant. Coimmunoprecipitation shows decreased association of STAT5b to MKL-1 TAD compared with WT-MKL-1; **d** The predicted MKL-1-binding site (STAT5b coiled-coil) is shown in **A**. This latter region was deleted to generate STAT5b coiled-coil domain mutant. Coimmunoprecipitation shows decreased association of MKL-1 to STAT5b coiled-coil compared with WT-STAT5b; **e** and **f** Luciferase assay detect expression of Foxp3 in HEK-293 T cells transfected with mutantion the coiled-coile domain of STAT5b and MKL-1 or the TAD domain of MKL-1 and STAT5b for 48 h. Results are normalized to the control or expressed as a percentage of the maximal effect of the given MKL-1 or STAT5b construct. **p < 0.01, *p < 0.05. Error bars indicate mean ± SEM

Deletion of the C-terminal domain (amino acids 715–931) to create Myocardin 1–715 leads to interfere with myocardial cell differentiation [25]. This mutant also abolished interactions between MKL-1and WT-STAT5b (Fig. 4a). This C-terminal domain of MKL-1 alone interacted directly with the WT-STAT5b (Fig. 4a). Our study support that the STAT5b interacts in vitro with MKL-1, and that the STAT5b interaction with MKL-1 is mediated through binding to its known C-terminal transactivation domain (TAD). We examined the functional domain of STAT5b involved in STAT5b-MKL-1 interaction in more detail by using a series of Flag-fusion STAT5b fragments, including Flag-STAT5b (amino acids 146–794); Flag-STAT5b (amino acids 332–794). As shown in Fig. 4b, in addition to the WT-STAT5b, the STAT5b activation domain (amino acids 146–794) was found to interact with MKL-1 in this assay, suggesting the possibility that the coiled-coil domains of STATA5A interact with MKL-1. Then, IL-2 enhances the interaction of MKL-1 and STAT5b (Fig. 4c and d). Mutated their interaction domain, this interaction did not exist (Fig. 4c and d), respectively. These data support that MKL-1TAD domain interacts with STAT5b coiled-coil domain. The mutation of TAD of MKL-1 resulted in an about 60% reduction in MKL-1 transactivation of the Foxp3 reporter. Similarly, the mutation of coiled-coil of STAT5b resulted in an about 70% reduction in STAT5b transactivation of the Foxp3 reporter (Fig. 4e and f). These findings imply that binding of STAT5 and MKL-1 is a critical mechanism in the MKL-1/STAT5b-mediated induction of the Foxp3 promoter.

### STAT5b strongly enhance the MKL-1 stimulation of the Foxp3 promoter and this effect requires the CArG box

**Fig. 5:**
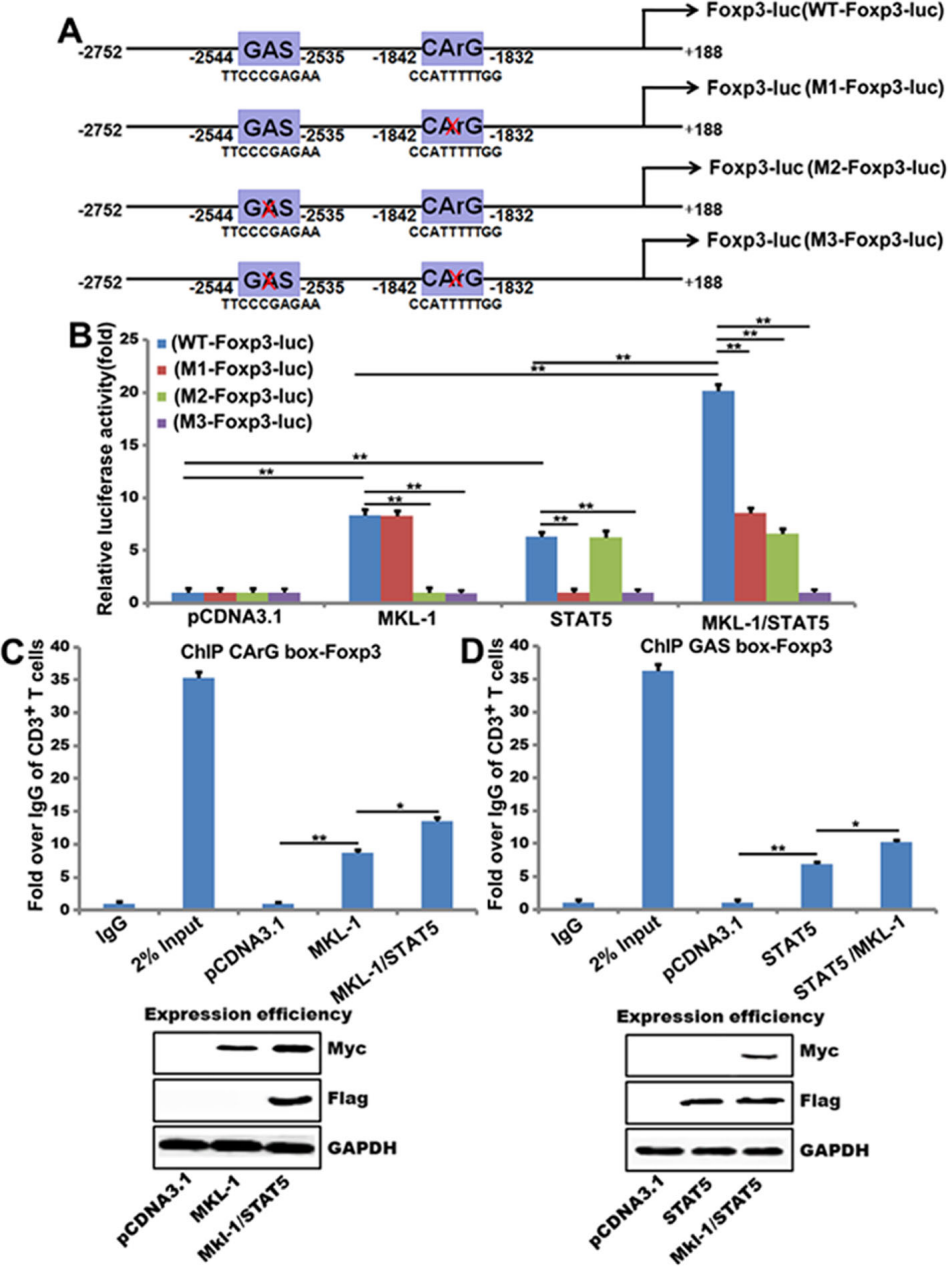
STAT5b strongly enhances the MKL-1 stimulation of the Foxp3 promoter and this effect requires the CArG box. **a** Schematic of the − 2752 Foxp3 promoter, containing CArG box and GAS elements were linked to a luciferase reporter. Mutation that remove the CArG box or GAS elements (M1-Foxp3-luc, M2-Foxp3-luc and M3-Foxp3-luc); **b** CD3^+^T cells were transfected with the Wild-Type-2752 Foxp3 promoter, or a M1-Foxp3-luc, M2-Foxp3-luc and M3-Foxp3-luc and transfected with MKL-1 or STAT5b for 48 h. Then the luciferase reporter assays was used to test the transactivity of Foxp3. (**p<0.01, *p<0.05) *n* = 3; **c** and **d** CD3^+^T cells were transiently transfected with a MKL-1, STAT5b, MKL-1 /STAT5b or a control vector (pCDNA3.1) 48 h, and ChIP assays were performed by PCR with primers associated with the genes for Foxp3. Sheared DNA/protein complexes were immunoprecipitated by using an anti-Myc-MKL-1 or anti-flag-STAT5b Ab. Then, PCR was carried out to detect the endogenous CArG regions or GAS regions in immunoprecipitated chromatin fragments. The amount of DNA in each sample (2% input) is shown at the second land. Immunoprecipitations were performed without primary antibody (No Ab) as a control and IgG as a negative control (**p<0.01) *n* = 3

Next, we sought to identify the critical promoter elements responsible for the effect of MKL-1, STAT5b and their synergy. The proximal portion of the Foxp3 promoter contains several regulatory elements, including one CArG box and one GAS (Fig. 5a). To characterize the importance of these elements, we generated a set of luciferase reporter constructs with various mutations of the Foxp3 promoter (Fig. 5a). In agreement with Supplementary Fig. 3D, both MKL-1 and STAT5b induced a modest increase in the promoter activity (8.2-fold and 5.9-fold, respectively), whereas the combined treatment acted synergistically (20-fold) (Fig. 5b). Interestingly, inactivating mutations of this element alone both inhibited the effect of the individual treatments and affected their synergy. Importantly, Mutation of the CArG box and GAS had an almost complete inhibitory effect (Fig. 5b). Together, these findings indicate that the CArG box and GAS are necessary and sufficient not only for Rho/Rho kinase-mediated activation of the promoter but also for the JAK-STAT5–triggered response and synergy between these signals.

ChIP assays further confirmed the impact of MKL-1/STAT5b on the Foxp3 gene promoters. Our results show that MKL-1 bind the CArG box of the Foxp3 promoter, STAT5b enhances this effect (Fig. 5c) and STAT5b bind the GAS of the Foxp3 promoter. Importantly, the binding ability of MKL-1/STAT5b on Foxp3 promoter is higher than the ability of STAT5b bind the GAS of the Foxp3 promoter (Fig. 5d). These results establish that the CArG box and GAS are necessary and sufficient for MKL-1 and STAT5b mediated Foxp3 promoter activity in Treg cells.

### STAT5b enhance the MKL-1-SRF interaction and induce MKL-1 to binding CArG box of Foxp3

**Fig. 6:**
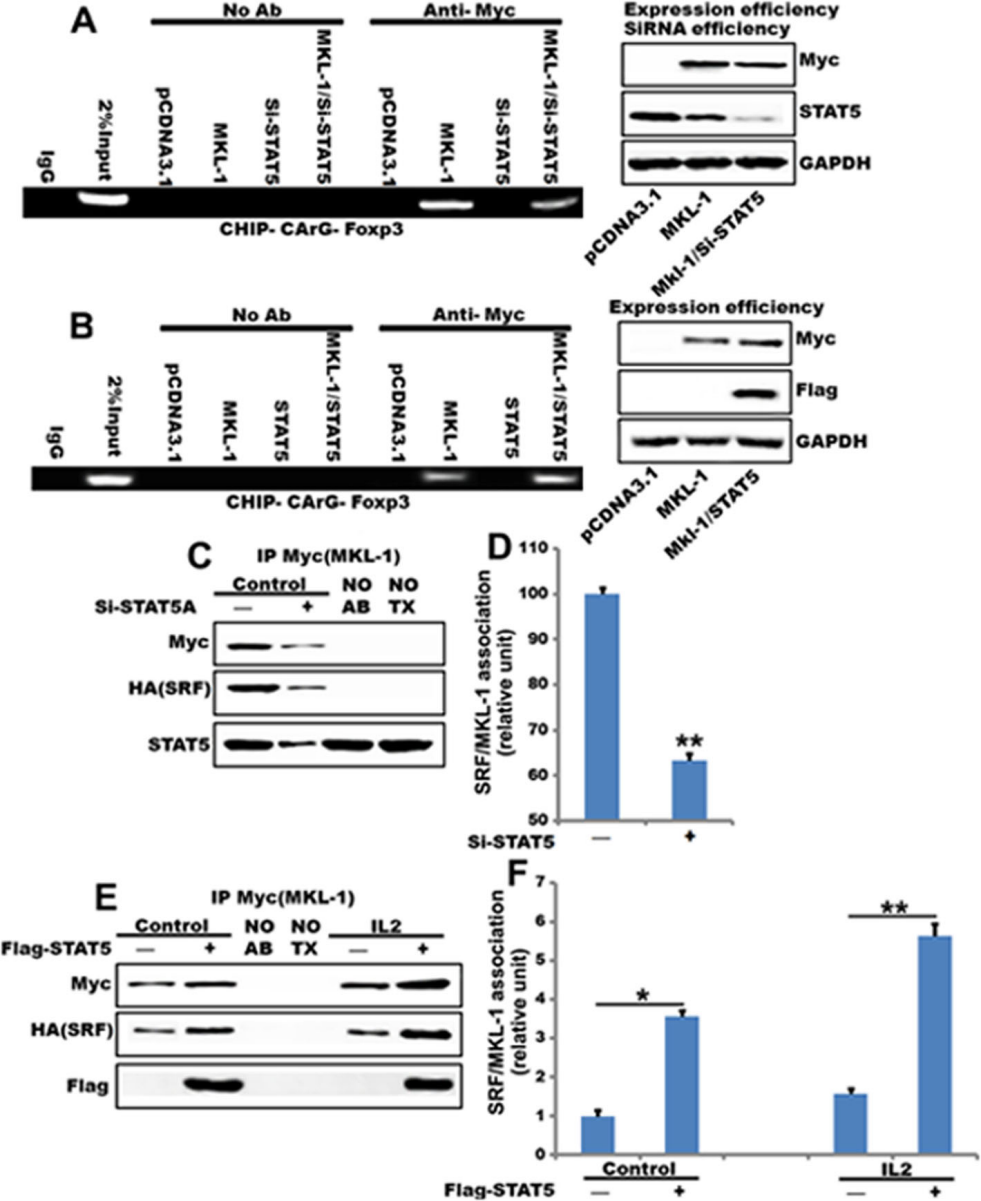
STAT5b enhances the MKL-1-SRF interaction and induces MKL-1 to binding CArG box of Foxp3. **a** and **b** CD3^+^T cells were transiently transfected with a MKL-1, siSTAT5b, STAT5b, MKL-1 /siSTAT5b or MKL-1 /STAT5b or a control vector (pCDNA3.1) 48 h, and ChIP assays were performed by PCR with primers associated with the genes for Foxp3 as described in Materials and Methods. Sheared DNA/protein complexes were immunoprecipitated by using an anti-Myc-MKL-1 Ab. Then, PCR was carried out to detect the endogenous CArG regions in immunoprecipitated chromatin fragments. The amount of DNA in each sample (2% input) is shown at the second land. Immunoprecipitations were performed without primary antibody (No Ab) as a control and IgG as a negative control (**p<0.01) *n* = 3; **c** Cells were transfected with Myc-MKL-1 and HA-SRF along with NR or STAT5b siRNA. Association of MKL-1 and SRF was analyzed by coimmunoprecipitation. STAT5b silencing was detected from whole cell lysates (WCL). Controls for the immunoprecipitation were reaction without antibody (No Ab) or Myc transfection (No Tx); **d** Densitometric analysis of three experiments is shown; **e** Myc-MKL-1 and HA-SRF were cotransfected with empty vector or STAT5b then treated IL-2. Coimmunoprecipitation shows IL-2 increased association of STAT5b to MKL-1 compared with DMSO. **f** Densitometric analysis of three experiments is shown

To assess the functional significance of MKL-1 and STAT5b, we tested whether STAT5b affects MKL-1-mediated transactivation of SRF-responsive, CArG-containing Foxp3. To examine the effect of STAT5b on MKL-1 transactivation in situ in CD3^+^ T cells, we used small RNA interference to diminish endogenous STAT5b expression or overexpression STAT5b and performed ChIP assays. The inhibition of STAT5b expression resulted in a reduction in MKL-1 binding ability of the Foxp3 reporter (Fig. 6a). In contrast, overexpression STAT5b resulted in an induction in MKL-1 binding ability of the Foxp3 reporter (Fig. 6b), supporting further that STAT5b is required for the complete transactivation of the, CArG-containing Foxp3 promoter by MKL-1. To gain insight into the molecular mechanism whereby STAT5b induce the function of MKL-1, we asked whether it interferes with the MKL-1–SRF interaction. To test this, we transfected cells with Myc-MKL-1 and HA-SRF and followed their association after silencing (Fig. 6c and d) or overexpressing STAT5b (Fig. 6e and f). The former condition strongly facilitated, whereas the latter markedly reduced the association of SRF with MKL-1. These findings imply that binding of STAT5b to MKL-1 is a critical mechanism in the MKL-1-mediated induction of the Foxp3 promoter.

### MKL-1, STAT5b, Foxp3 and CD25 have lower expression in ITP patients

**Fig. 7:**
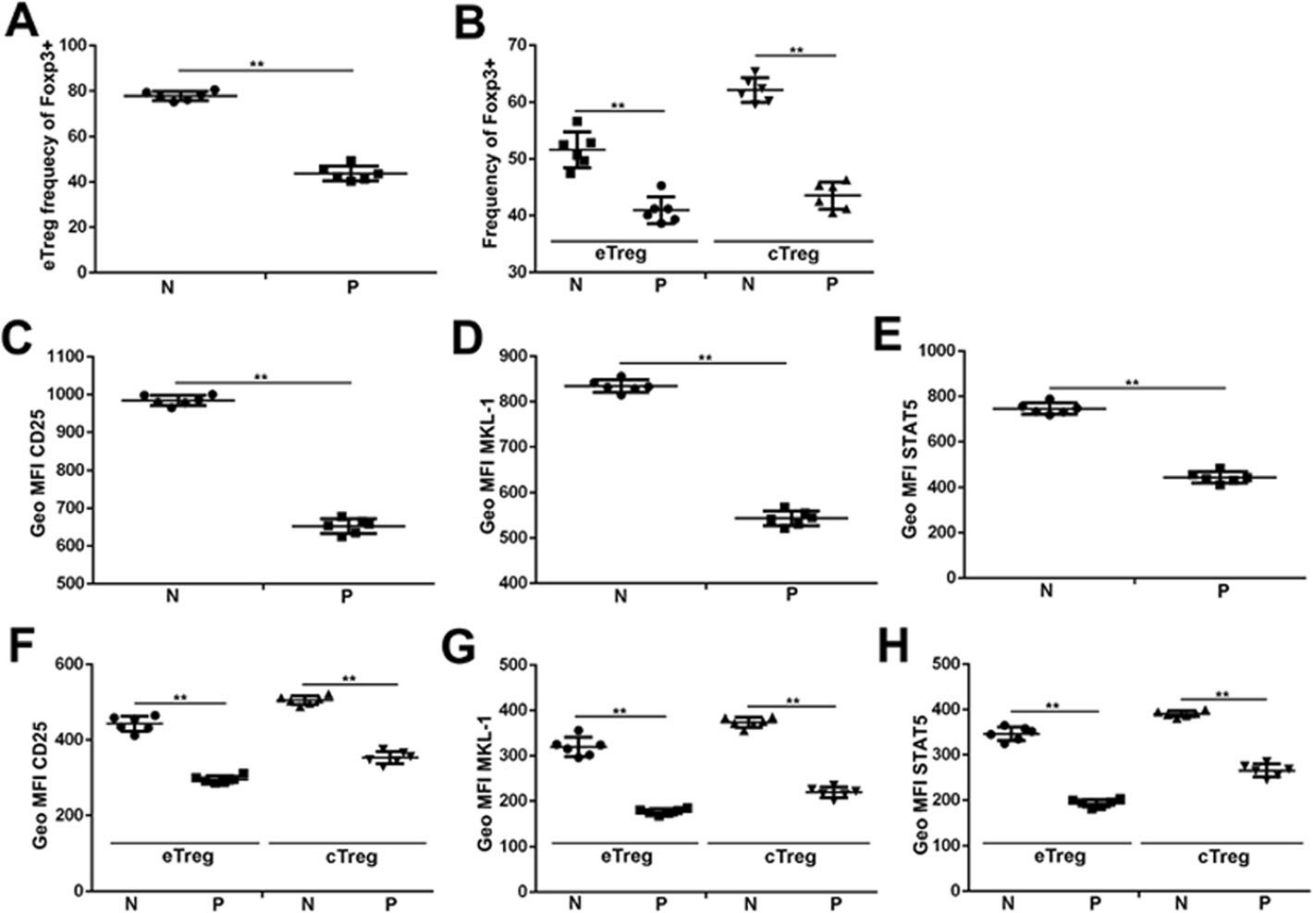
MKL-1, STAT5b, Foxp3 and CD25 have lower expression in ITP patients. MKL-1, STAT5b, Foxp3 and CD25 expression was examined by qPCR in ITP patients. GAPDH was used as a loading control. **p < 0.01. *n* = 6

Recently, Treg are a heterogeneous population that shows a high degree of phenotypic and functional specialization defined as cTreg (CD44^low^CD62L^high^) and eTreg (CD44^high^CD62L^low^). To understand whether RhoA-MKL-1 and JAK-STAT5b signaling facilitates differentiation of eTreg in ITP patients, we analyzed Treg subsets both normal persons and ITP patients. Interestingly the frequency of eTreg was specifically decreased in ITP patients (Fig. 7a). We confirmed that eTreg have lower Foxp3 expression than cTreg in both normal persons and ITP patients (Fig. 7b). As shown in Fig. 7c-e, ITP patients have lower the expression of MKL-1, STAT5b and CD25 than normal persons. Moreover, eTreg have lower MKL-1, STAT5b and CD25 expression than cTreg in both normal persons and ITP patients (Fig. 7f-h). Collectively, these data suggest that MKL-1, STAT5b, Foxp3 and CD25 have lower expression in ITP patients and ITP patients have lower the frequency of eTreg.

## Discussion

CD4^+^CD25^+^Foxp3^+^ Tregs that develop in the thymus constitute 2–4% of CD4 single positive thymocytes, yet this relatively small population plays a critical role in maintaining peripheral tolerance and preventing autoimmunity.

MKL-1, a cofactor of SRF, is a SAP (SAF-A/B, acinus, PIAS) domain containing protein widely expressed in various tissues [26, 27]. MKL1 inhibits tumor necrosis factor induced cell death in embryonic fibroblasts [28]. Our previous reports that MKL-1 and STAT3 synergistically promote breast cancer cell migration and via hypermethylating BRSM1 [29, 30]. Recent reports that SRF and its transcriptional cofactor MKL1 are critical for megakaryocyte maturation and platelet formation [31], which may be relevant to its role in Treg cell phenotype and function. Our data presented here demonstrate a novel role for MKL-1 in regulating Treg cell phenotype and function. We show that MKL-1 expression is up-regulated during Treg cell differentiation and that enforced expression of MKL-1 in CD3^+^ T cells enhances Treg markers expression. Importantly, here we show that MKL-1 plays a role in Treg cell differentiation, and have begun to elucidate the mechanisms underlying MKL1-mediated Treg cell phenotype and function.

Despite 96% homology, STAT5a and STAT5b play distinct roles during development [32], STAT5b knockout alone caused retarded growth, as in the Laron dwarfism syndrome [33]. Although lack of STAT5a or STAT5b has no impact on the immune response, double STAT5a/b deficiency caused impaired proliferation in response to IL-2 and halted cell cycle progression of mature T cells [34]. STAT5a/b transcription factors protect the survival of activated T cells [35]. IL-2/STAT5-dependent signaling may be important to control self-tolerance by Treg cells [36]. Interestingly, IL-2R/STAT5 signaling also influences selection of the thymic Treg TCR repertoire [37]. Another recent study also examined the role of CD4^+^CD25^+^ Treg cells in inhibiting autoreactive T cells [38]. Signalling from IL-2RG via Janus kinase 3 (JAK3) leads to signal transducer and activator of transcription-5 (STAT-5) activation [39], and is needed for T cell proliferation and differentiation and expression of anti-apoptotic molecules [34, 40].

The molecular mechanism by which STAT5 affects Foxp3 transcription is also unclear. STAT5 binding sites have been found in the Foxp3 promoter region as well as within the CNS2 region of intron 1 in the Foxp3 gene and several studies have shown STAT5 binding to those sites [14, 15]. Deletion of the entire CNS2 region including the STAT5 binding sites did not prevent Treg development although it did have an effect on stability of Foxp3 expression [41]. The effect of STAT5 binding to these sites is not yet clear. Our findings indicate that the CArG box and GAS are necessary and sufficient not only for Rho/Rho kinase-mediated activation of the promoter but also for the JAK-STAT5–triggered response and synergy between these signals.

The critical co-factors that interact with STAT5 to promote Treg development are also poorly characterized. STAT5 is known to interact with a variety of both co-activators, such as CBP and p300, and co-repressors such as NCOR2 [42, 43]. How these function in Treg differentiation remains untested. Intriguingly, treatment of Treg progenitors with two distinct histone deactylase (HDAC) inhibitors prevented the IL-2/STAT5-dependent conversion of Treg progenitors into Tregs [44]. Our studies demonstrate for the first time that MKL-1 is a coactivator for STAT5b, the regulator of Treg cell development and function.

Clinical efficacy has been demonstrated with antibodies blocking inhibitory immune checkpoints such as cytotoxic T lymphocyte-associated antigen 4 (CTLA-4) or PD-1 / PD-L1, its mechanism of action is, however, only partially understood, anti-CTLA- 4 therapy may target Tregs in vivo, and CTLA4 / PKCη signaling pathway that we recently found to be required for contact-dependent Treg-mediated suppression [45, 46].

## Conclusions

Collectively, we demonstrate the pivotal role of the STAT5b–MKL-1 interaction identifies a role of Treg-specific gene regulation and regulated Treg cell development and function and suggests a possible mechanism for the protective effects of autoimmune disease ITP.

## Supplementary information

**Additional file 1: Figure S1.** Over-expression MKL-1 and STAT5b increase the number of Treg in CD3^+^ T cells and enhance the Treg markers expression. **A**. Western blot analysis of MKL-1 and STAT5b protein level in CD3^+^T cells transfected with myc-MKL-1 or flag-STAT5b for 48 h. **B**. The number of Treg in CD3^+^T cells transfected with myc-MKL-1 or flag-STAT5b for 48 h by flow cytometry. **C**. QPCR analysis of Foxp3 and CD25 mRNA level in CD3^+^T cells transfected with myc-MKL-1 or flag-STAT5b for 48 h. GAPDH is the loading control. **, *P* < 0.01, *, *P* < 0.05. *n* = 3. **D** and **E.** Western blot analysis of Foxp3 and CD25 protein level in CD3^+^T cells transfected with myc-MKL-1 or flag-STAT5b for 48 h. Data were quantified using Quantity One software. GAPDH is the loading control. **, *P* < 0.01, *, *P* < 0.05. *n* = 3. **Figure S2.** Inhibited or knock-down MKL-1 and STAT5b weaken the Treg markers expression. A. QPCR analysis of Foxp3 and CD25 mRNA level in CD3^+^T cells treated with AG490 or Y27632 for 48 h. GAPDH is the loading control. **, *P* < 0.01, *, *P* < 0.05. *n* = 3. B. QPCR analysis of Foxp3 and CD25 mRNA level in CD3^+^T cells transfected with MKL-1 and STAT5b siRNA for 48 h. GAPDH is the loading control. **, *P* < 0.01, *, *P* < 0.05. *n* = 3. C and E. Western blot analysis of Foxp3 and CD25 mRNA level in CD3^+^T cells treated with AG490 or Y27632 for 48 h. Data were quantified using Quantity One software. GAPDH is the loading control. **, *P* < 0.01, *, *P* < 0.05. *n* = 3. D and F. Western blot analysis of Foxp3 and CD25 protein level in CD3^+^T cells transfected with MKL-1 and STAT5b siRNA for 48 h. Data were quantified using Quantity One software. GAPDH is the loading control. **, *P* < 0.01, *, *P* < 0.05. *n* = 3. **Figure S3.** IL2 affects the effect MKL-1 and STAT5b on the Treg marker expression. A. QPCR analysis of Foxp3 protein level in CD3^+^T cells transfected with MKL-1 and STAT5b and treated with IL2 for 48 h. GAPDH is the loading control. **, *P* < 0.01, *, *P* < 0.05. *n* = 3. B and C. Western blot analysis of Foxp3 protein level in CD3^+^T cells transfected with MKL-1 and STAT5b and treated with IL2 for 48 h. Data were quantified using Quantity One software. GAPDH is the loading control. **, *P* < 0.01, *, *P* < 0.05. *n* = 3. D. The luciferase reporter assays were used to test the transactivity of Foxp3 in CD3^+^T cells transfected with MKL-1 and STAT5b and treated with IL2 for 48 h. **, *P* < 0.01, *, *P* < 0.05, *n* = 6. **Figure S4.** Ag490 and Y27632 affect the phosphorylation of Foxp3 and nuclear accumulation of Foxp3. A and B. Western blot analysis to detect phosphorylated Foxp3 in CD3^+^T cells treated with AG490 or Y27632 for 48 h. Data were quantified using Quantity One software. GAPDH is the loading control. **, *P* < 0.01, *, *P* < 0.05. *n* = 3. C and D. Western blot analysis to detect nuclear or membrane Foxp3 in CD3 + T cells treated with AG490 or Y27632 for 48 h. Data were quantified using Quantity One software. GAPDH is the loading control. **, *P* < 0.01, *, *P* < 0.05. *n* = 3.

## Data Availability

The data generated during this study are included in this article and its supplementary information files are available from the corresponding author on reasonable request.

## Abbreviations

Treg: Foxp3^+^CD4^+^ regulatory T cells
ITP: Idiopathic Thrombocytopenic Purpura
MKL-1: Megakaryoblastic leukemia 1
SRF: Serum response factor
STAT: The signal transducer and activator of transcription
IL-2: Interleukin-2
FITC: Fluorescein isothiocyanate
DAPI: 4′,6-diamidino-2-phenylindole
qRT-PCR: Quantitative real-time PCR
NC: Negative control

## References

[1] Schmaler M, Broggi MA, Lagarde N, Stöcklin BF, King CG, Finke D, Rossi SW (2015). IL-7R signaling in regulatory T cells maintains peripheral and allograft tolerance in mice. Proc Natl Acad Sci U S A.

[2] Yu J, Heck S, Patel V, Levan J, Yu Y, Bussel JB, Yazdanbakhsh K (2008). Defective circulating CD25 regulatory T cells in patients with chronic immune thrombocytopenic purpura. Blood.

[3] Ma Z, Morris SW, Valentine V, Li M, Herbrick JA, Cui X, Bouman D, Li Y, Mehta PK, Nizetic D (2001). Fusion of two novel genes, RBM15 and MKL1, in the t(1;22)(p13;q13) of acute megakaryoblastic leukemia. Nat Genet.

[4] Mercher T, Coniat MB, Monni R, Mauchauffe M, Nguyen KF, Gressin L, Mugneret F, Leblanc T, Dastugue N, Berger R (2001). Involvement of a human gene related to the Drosophila spen gene in the recurrent t(1;22) translocation of acute megakaryocytic leukemia. Proc Natl Acad Sci U S A.

[5] Wang DZ, Li S, Hockemeyer D, Sutherland L, Wang Z, Schratt G, Richardson JA, Nordheim A, Olson EN (2002). Potentiation of serum response factor activity by a family of myocardin-related transcription factors. Proc Natl Acad Sci U S A.

[6] Muehlich S, Wang R, Lee SM, Lewis TC, Dai C, Prywes R (2008). Serum-induced phosphorylation of the serum response factor coactivator MKL1 by the extracellular signal-regulated kinase 1/2 pathway inhibits its nuclear localization. Mol Cell Biol.

[7] Cen B, Selvaraj A, Burgess RC, Hitzler JK, Ma Z, Morris SW, Prywes R (2003). Megakaryoblastic leukemia 1, a potent transcriptional coactivator for serum response factor (SRF), is required for serum induction of SRF target genes. Mol Cell Biol.

[8] Du KL, Chen M, Li J, Lepore JJ, Mericko P, Parmacek MS (2004). Megakaryoblastic leukemia factor-1 transduces cytoskeletal signals and induces smooth muscle cell differentiation from undifferentiated embryonic stem cells. J Biol Chem.

[9] Tabuchi A, Estevez M, Henderson JA, Marx R, Shiota J, Nakano H, Baraban JM (2010). Nuclear translocation of the SRF co-activator MAL in cortical neurons: role of RhoA signalling. J Neurochem.

[10] Halene S, Gao Y, Hahn K, Massaro S, Italiano JE, Schulz V, Lin S, Kupfer GM, Krause DS (2010). Serum response factor is an essential transcription factor in megakaryocytic maturation. Blood.

[11] Buggins AGS, Pepper CJ (2010). The role of Bcl-2 family proteins in chronic lymphocytic leukaemia. Leuk Res.

[12] Nadeau K, Hwa V, Rosenfeld RG (2011). STAT5b deficiency: an unsuspected cause of growth failure, immunodeficiency, and severe pulmonary disease. J Pediatr.

[13] Cohen AC, Nadeau KC, Tu W, Hwa V, Dionis K, Bezrodnik L, Teper A, Gaillard M, Heinrich J, Krensky AM (2006). Cutting edge: decreased accumulation and regulatory function of CD4+CD25high T cells in human STAT5b deficiency. J Immunol.

[14] Burchill MA, Yang J, Vogtenhuber C, Blazar BR, Farrar MA (2007). IL-2 receptor β-dependent STAT5 activation is required for the development of Foxp3+ regulatory T cells. J Immunol.

[15] Yao Z, Kanno Y, Kerenyi M, Stephens G, Durant L, Watford WT, Laurence A, Robinson GW, Shevach EM, Moriggl R (2007). Nonredundant roles for Stat5a/b in directly regulating Foxp3. Blood.

[16] Jeffery HC, Jeffery LE, Lutz P, Corrigan M, Webb GJ, Hirschfield GM, Adams DH, Oo YH (2017). Low-dose interleukin-2 promotes STAT-5 phosphorylation, Tregsurvival and CTLA-4-dependent function in autoimmune liver diseases. Clin Exp Immunol.

[17] Fontenot JD, Gavin MA, Rudensky AY (2003). Foxp3 programs the development and function of CD4+CD25+ regulatory T cells. Nat Immunol.

[18] Wieczorek G, Asemissen A, Model F, Turbachova I, Floess S, Liebenberg V, Baron U, Stauch D, Kotsch K, Pratschke J (2009). Quantitative DNA methylation analysis of FOXP3 as a new method for counting regulatory T cells in peripheral blood and solid tissue. Cancer Res.

[19] Hoffmann P, Eder R, Boeld TJ, Doser K, Piseshka B, Andreesen R, Edinger M (2006). Only the CD45RA+ subpopulation of CD4+CD25high T cells gives rise to homogeneous regulatory T-cell lines upon in vitro expansion. Blood.

[20] Battaglia M, Stabilini A, Roncarolo MG (2005). Rapamycin selectively expands CD4+CD25+FoxP3+ regulatory T cells. Blood.

[21] Okubo Y, Torrey H, Butterworth J, Zheng H, Faustman DL (2016). Treg activation defect in type 1 diabetes: correction with TNFR2 agonism. Clin Transl Immunol.

[22] Zhu J, Shevach EM (2014). TCR signaling fuels Treg cell suppressor function. Nat Immunol.

[23] Liao XH, Lu DL, Wang N, Liu LY, Wang Y, Li YQ, Yan TB, Sun XG, Hu P, Zhang TC (2014). Estrogen receptor α mediates proliferation of breast cancer MCF-7 cells via a p21/PCNA/E2F1-dependent pathway. FEBS J.

[24] Sadlack B, Merz H, Schorle H, Schimpl A, Feller AC, Horak I (1993). Ulcerative colitis-like disease in mice with a disrupted interleukin-2 gene. Cell.

[25] Wang L, Di TM, Beecham A, Slifer S, Rundek T, Homma S, Blanton SH, Sacco RL (2010). A comprehensive genetic study on left atrium size in Caribbean Hispanics identifies potential candidate genes in 17p10. Circ Cardiovasc Genet.

[26] Brandt DT, Xu J, Steinbeisser H, Grosse R (2009). Regulation of myocardin-related transcriptional coactivators through cofactor interactions in differentiation and cancer. Cell Cycle.

[27] Selvaraj A, Prywes R (2004). Expression profiling of serum inducible genes identifies a subset of SRF target genes that are MKL dependent. BMC Mol Biol.

[28] Sasazuki T, Sawada T, Sakon S, Kitamura T, Kishi T, Okazaki T, Katano M, Tanaka M, Watanabe M, Yagita H (2002). Identification of a novel transcriptional activator, BSAC, by a functional cloning to inhibit tumor necrosis factor-induced cell death. J Biol Chem.

[29] Wen-Jing X, Xing-Hua L, Nan W, Dong-Wei Z, Li Z, De-Liang Z, Jian D, Tong-Cun Z (2015). MRTF-A and STAT3 promote MDA-MB-231 cell migration via hypermethylating BRSM1. IUBMB Life.

[30] Liao XH, Wang N, Liu LY, Zheng L, Xing WJ, Zhao DW, Sun XG, Hu P, Dong J, Zhang TC (2014). MRTF-A and STAT3 synergistically promote breast cancer cell migration. Cell Signal.

[31] Smith EC, Thon JN, Devine MT, Lin S, Schulz VP, Guo Y, Massaro SA, Halene S, Gallagher P (2012). IJ: MKL1 and MKL2 play redundant and crucial roles in megakaryocyte maturation and platelet formation. Blood.

[32] Liu X, Robinson GW, Wagner KU, Garrett L, Wynshawboris A, Hennighausen L (1997). Stat5a is mandatory for adult mammary gland development and lactogenesis. Genes Dev.

[33] Udy GB, Towers RP, Snell RG, Wilkins RJ, Park SH, Ram PA, Waxman DJ, Davey HW (1997). Requirement of STAT5b for sexual dimorphism of body growth rates and liver gene expression. Proc Natl Acad Sci U S A.

[34] Moriggl R, Topham DJ, Teglund S, Sexl V, Mckay C, Wang D, Hoffmeyer A, Deursen JV, Sangster MY, Bunting KD (1999). Stat5 is required for IL-2-induced cell cycle progression of peripheral T cells. Immunity.

[35] Zhang Y, Kirken RA, Furian L, Janczewska S, Qu X, Hancock WW, Wang M, Tejpal N, Kerman R, Kahan BD (2006). Allograft rejection requires STAT5a/b-regulated antiapoptotic activity in T cells but not B cells. J Immunol.

[36] Kieslinger M, Woldman I, Moriggl R, Hofmann J, Marine JC, Ihle JN, Beug H, Decker T (2000). Antiapoptotic activity of STAT5 required during terminal stages of myeloid differentiation. Genes Dev.

[37] Mahmud SA, Manlove LS, Farrar MA (2013). Interleukin-2 and STAT5 in regulatory T cell development and function. JAK-STAT.

[38] Antov A, Yang L, Vig M, Baltimore D, Parijs LV (2003). Essential role for STAT5 signaling in CD25+CD4+ regulatory T cell homeostasis and the maintenance of self-tolerance. J Immunol.

[39] Kirken RA, Rui H, Malabarba GM, Howard ZOM, Kawamura M, O'Shea JJ, Farrar WL (1995). Activation of JAK3, but not JAK1, is critical for IL-2-induced proliferation and STAT5 recruitment by a COOH-terminal region of the IL-2 receptor β-chain. Cytokine.

[40] Lord JD, Mcintosh BC, Greenberg PD, Nelson BH (2000). The IL-2 receptor promotes lymphocyte proliferation and induction of the c-myc, bcl-2, and bcl-x genes through the trans-activation domain of Stat5. J Immunol.

[41] Zheng Y, Josefowicz S, Chaudhry A, Peng XP, Forbush K, Rudensky AY (2010). Role of conserved non-coding DNA elements in the Foxp3 gene in regulatory T-cell fate. Nature.

[42] Pfitzner E, Jähne R, Wissler M, Stoecklin E, Groner B (1998). p300/CREB-binding protein enhances the prolactin-mediated transcriptional induction through direct interaction with the transactivation domain of Stat5, but does not participate in the Stat5-mediated suppression of the glucocorticoid response. Mol Endocrinol.

[43] Nakajima H, Brindle P, Handa M, Ihle J (2014). Functional interaction of STAT5 and nuclear receptor co-repressor SMRT: implications in negative regulation of STAT5-dependent transcription. EMBO J.

[44] Burchill MA, Yang J, Vang KB, Moon JJ, Chu HH, Lio CW, Vegoe AL, Hsieh CS, Jenkins MK, Farrar MA (2008). Linked T cell receptor and cytokine signaling govern the development of the regulatory T cell repertoire. Immunity.

[45] Romano E, Kusiokobialka M, Foukas PG, Baumgaertner P, Meyer C, Ballabeni P, Michielin O, Weide B, Romero P, Speiser DE (2015). Ipilimumab-dependent cell-mediated cytotoxicity of regulatory T cells ex vivo by nonclassical monocytes in melanoma patients. Proc Natl Acad Sci U S A.

[46] Pedros C, Canonigobalancio AJ, Kong K, Altman A (2017). Requirement of Treg-intrinsic CTLA4/PKCη signaling pathway for suppressing tumor immunity. JCI insight.

