# Supplementary material for: MKL-1 is a coactivator for STAT5b, the regulator of Treg cell development and function
Source: Cell Commun Signal. 2020 Jul 9;18:107. doi: 10.1186/s12964-020-00574-1 (PMC7350762; doi:10.1186/s12964-020-00574-1)
Supplement: Supplementary file 2 — Additional file 1: Figure S1. Over-expression MKL-1 and STAT5b increase the number of Treg in CD3+ T cells and enhance the Treg markers expression. A. Western blot analysis of MKL-1 and STAT5b protein level in CD3+T cells transfected with myc-MKL-1 or flag-STAT5b for 48 h. B. The number of Treg in CD3+T cells transfected with myc-MKL-1 or flag-STAT5b for 48 h by flow cytometry. C. QPCR analysis of Foxp3 and CD25 mRNA level in CD3+T cells transfected with myc-MKL-1 or flag-STAT5b for 48 h. GAPDH is the loading control. **, P < 0.01, *, P < 0.05. n = 3. D and E. Western blot analysis of Foxp3 and CD25 protein level in CD3+T cells transfected with myc-MKL-1 or flag-STAT5b for 48 h. Data were quantified using Quantity One software. GAPDH is the loading control. **, P < 0.01, *, P < 0.05. n = 3. Figure S2. Inhibited or knock-down MKL-1 and STAT5b weaken the Treg markers expression. A. QPCR analysis of Foxp3 and CD25 mRNA level in CD3+T cells treated with AG490 or Y27632 for 48 h. GAPDH is the loading control. **, P < 0.01, *, P < 0.05. n = 3. B. QPCR analysis of Foxp3 and CD25 mRNA level in CD3+T cells transfected with MKL-1 and STAT5b siRNA for 48 h. GAPDH is the loading control. **, P < 0.01, *, P < 0.05. n = 3. C and E. Western blot analysis of Foxp3 and CD25 mRNA level in CD3+T cells treated with AG490 or Y27632 for 48 h. Data were quantified using Quantity One software. GAPDH is the loading control. **, P < 0.01, *, P < 0.05. n = 3. D and F. Western blot analysis of Foxp3 and CD25 protein level in CD3+T cells transfected with MKL-1 and STAT5b siRNA for 48 h. Data were quantified using Quantity One software. GAPDH is the loading control. **, P < 0.01, *, P < 0.05. n = 3. Figure S3. IL2 affects the effect MKL-1 and STAT5b on the Treg marker expression. A. QPCR analysis of Foxp3 protein level in CD3+T cells transfected with MKL-1 and STAT5b and treated with IL2 for 48 h. GAPDH is the loading control. **, P < 0.01, *, P < 0.05. n = 3. B and C. Western blot analysis of F [file 12964_2020_574_MOESM2_ESM.docx]

Supplemental material

**
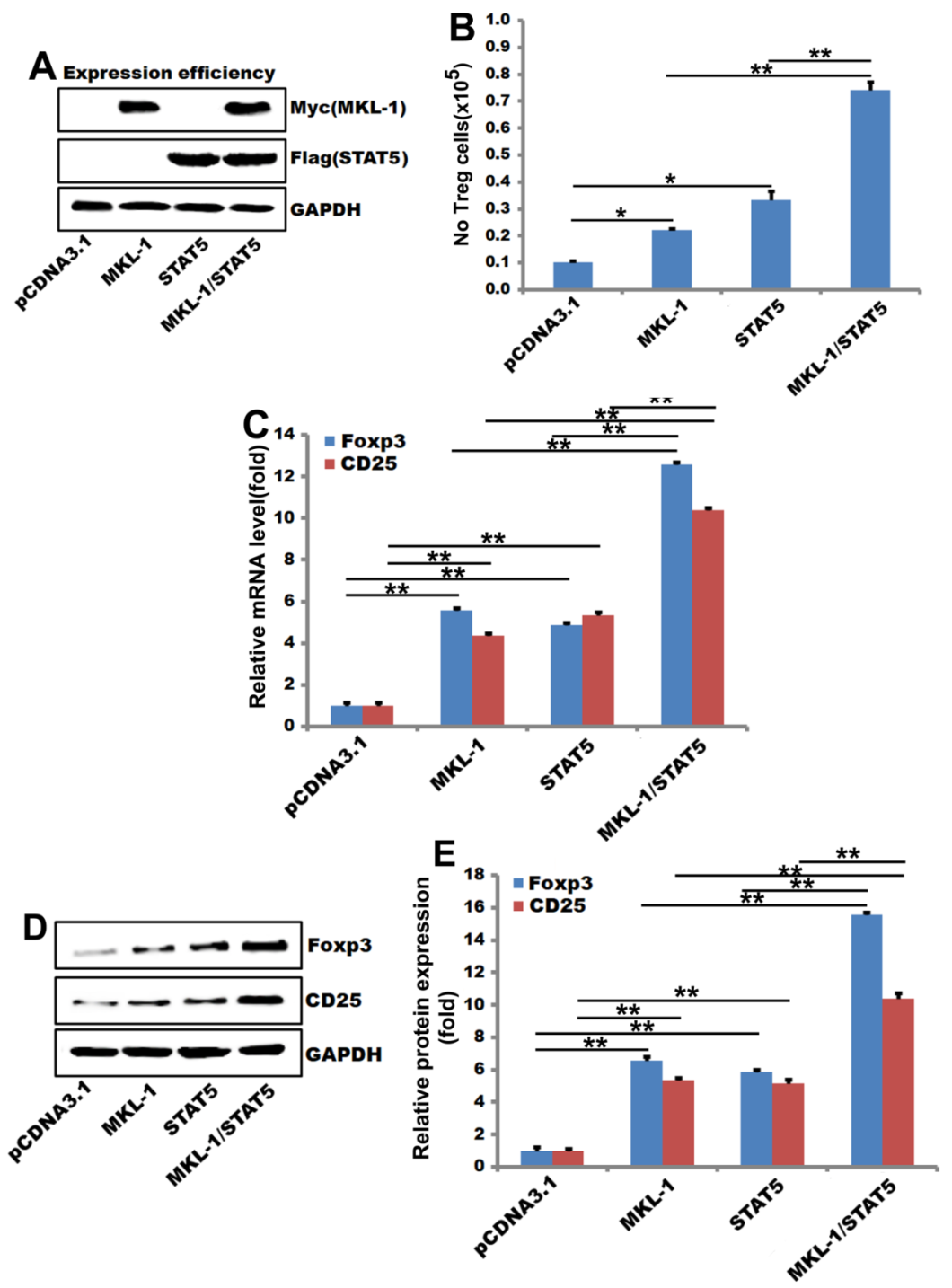
**

**Figure S1.** Over-expression MKL-1 and STAT5b increase the number of Treg in CD3^+^ T cells and enhance the Treg markers expression.

**A**. Western blot analysis of MKL-1 and STAT5b protein level in CD3^+^T cells transfected with myc-MKL-1 or flag-STAT5b for 48 hours.

**B**. The number of Treg in CD3^+^T cells transfected with myc-MKL-1 or flag-STAT5b for 48 hours by flow cytometry.

**C**. QPCR analysis of Foxp3 and CD25 mRNA level in CD3^+^T cells transfected with myc-MKL-1 or flag-STAT5b for 48 hours. GAPDH is the loading control. **, *P*<0.01, *, *P*<0.05. n=3.

**D** and **E.** Western blot analysis of Foxp3 and CD25 protein level in CD3^+^T cells transfected with myc-MKL-1 or flag-STAT5b for 48 hours. Data were quantified using Quantity One software. GAPDH is the loading control. **, *P*<0.01, *, *P*<0.05. n=3.


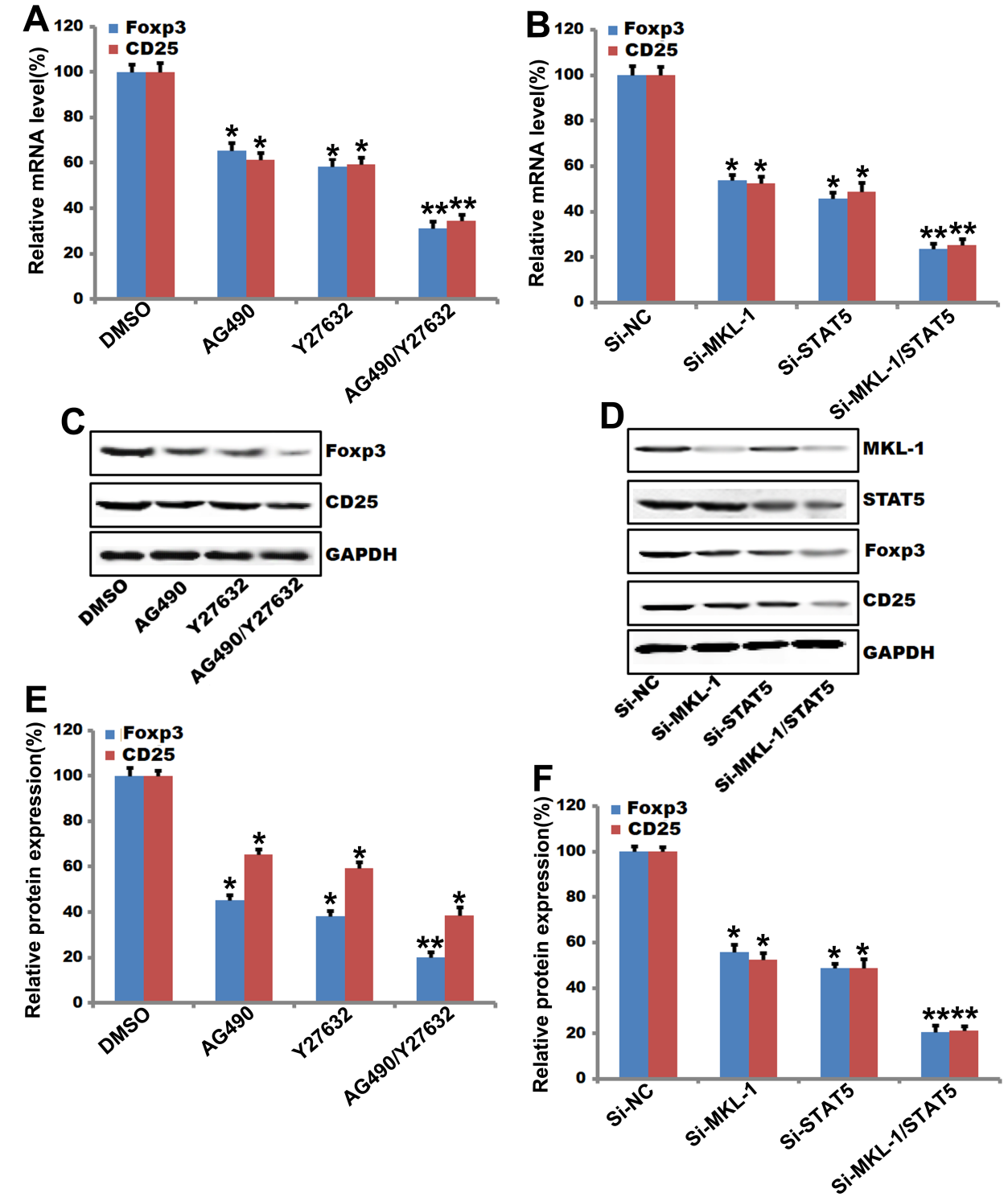


**Figure S2.** Inhibited or knock-down MKL-1 and STAT5b weaken the Treg markers expression.

A. QPCR analysis of Foxp3 and CD25 mRNA level in CD3^+^T cells treated with AG490 or Y27632 for 48 hours. GAPDH is the loading control. **, *P*<0.01, *, *P*<0.05. n=3.

B. QPCR analysis of Foxp3 and CD25 mRNA level in CD3^+^T cells transfected with MKL-1 and STAT5b siRNA for 48 hours. GAPDH is the loading control. **, *P*<0.01, *, *P*<0.05. n=3.

C and E. Western blot analysis of Foxp3 and CD25 mRNA level in CD3^+^T cells treated with AG490 or Y27632 for 48 hours. Data were quantified using Quantity One software. GAPDH is the loading control. **, *P*<0.01, *, *P*<0.05. n=3.

D and F. Western blot analysis of Foxp3 and CD25 protein level in CD3^+^T cells transfected with MKL-1 and STAT5b siRNA for 48 hours. Data were quantified using Quantity One software. GAPDH is the loading control. **, *P*<0.01, *, *P*<0.05. n=3.

**
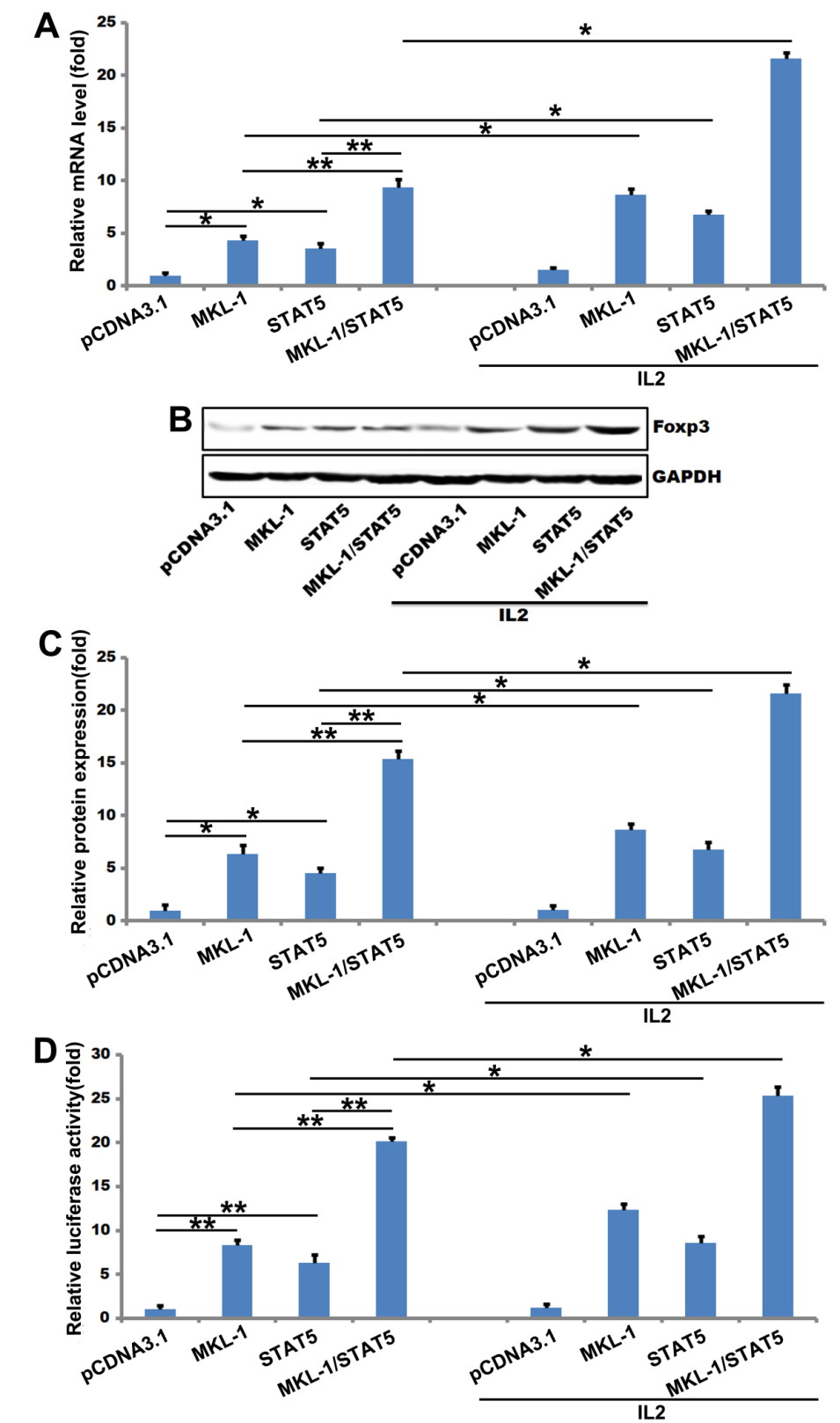
**

**Figure S3.** IL2 affects the effect MKL-1 and STAT5b on the Treg marker expression.

A. QPCR analysis of Foxp3 protein level in CD3^+^T cells transfected with MKL-1 and STAT5b and treated with IL2 for 48 hours. GAPDH is the loading control. **, *P*<0.01, *, *P*<0.05.n=3.

B and C. Western blot analysis of Foxp3 protein level in CD3^+^T cells transfected with MKL-1 and STAT5b and treated with IL2 for 48 hours. Data were quantified using Quantity One software. GAPDH is the loading control. **, *P*<0.01, *, *P*<0.05. n=3.

D. The luciferase reporter assays were used to test the transactivity of Foxp3 in CD3^+^T cells transfected with MKL-1 and STAT5b and treated with IL2 for 48 hours. **, *P*<0.01, *, *P*<0.05, n=6.

**
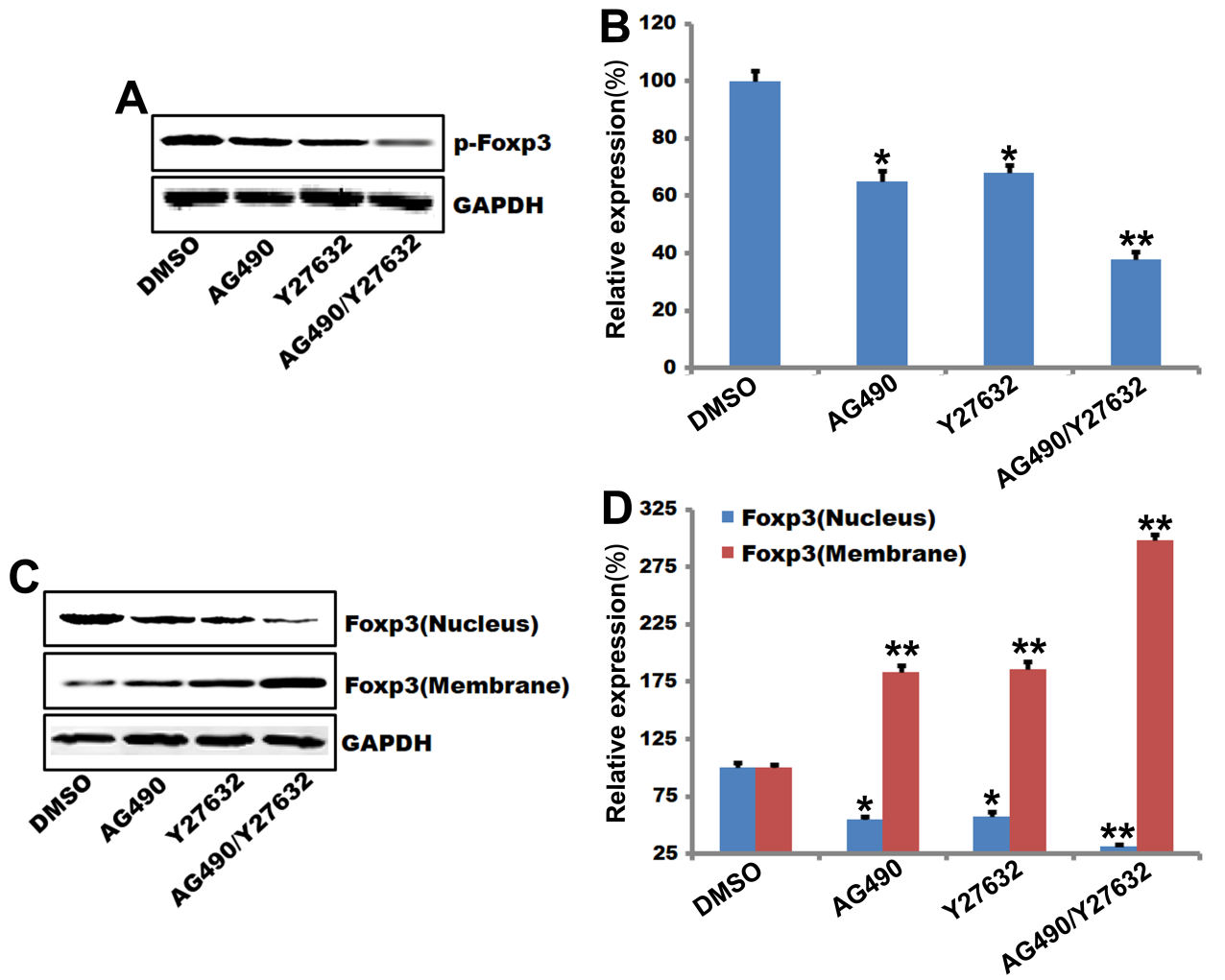
**

**Figure S4.** Ag490 and Y27632 affect the phosphorylation of Foxp3 and nuclear accumulation of Foxp3.

A and B. Western blot analysis to detect phosphorylated Foxp3 in CD3^+^T cells treated with AG490 or Y27632 for 48 hours. Data were quantified using Quantity One software. GAPDH is the loading control. **, *P*<0.01, *, *P*<0.05. n=3.

C and D. Western blot analysis to detect nuclear or membrane Foxp3 in CD3+T cells treated with AG490 or Y27632 for 48 hours. Data were quantified using Quantity One software. GAPDH is the loading control. **, *P*<0.01, *, *P*<0.05. n=3.
